# Mechanistic insights into the formation of hydroxides with unconventional coordination environments to achieve their cost-effective synthesis

**DOI:** 10.1093/nsr/nwae427

**Published:** 2024-12-23

**Authors:** Zongkun Chen, Qiqi Fan, Xingkun Wang, Liqun Kang, Wenchao Wan, Jonathan Thomas Avaro, Siyuan Zhang, Christina Scheu, Jian Zhou, Serena DeBeer, Saskia Heumann, Minghua Huang, Heqing Jiang, Helmut Cölfen

**Affiliations:** Max Planck Institute for Chemical Energy Conversion, Mülheim an der Ruhr 45470, Germany; University of Konstanz, Physical Chemistry, Konstanz 78457, Germany; University of Konstanz, Physical Chemistry, Konstanz 78457, Germany; Qingdao New Energy Shandong Laboratory, Qingdao Institute of Bioenergy and Bioprocess Technology, Chinese Academy of Sciences, Qingdao 266101, China; School of Materials Science and Engineering, Ocean University of China, Qingdao 266100, China; Max Planck Institute for Chemical Energy Conversion, Mülheim an der Ruhr 45470, Germany; Max Planck Institute for Chemical Energy Conversion, Mülheim an der Ruhr 45470, Germany; EMPA—Materials Science and Technology, St. Gallen 9014, Switzerland; Max-Planck-Institut für Eisenforschung GmbH, Düsseldorf 40237, Germany; Max-Planck-Institut für Eisenforschung GmbH, Düsseldorf 40237, Germany; University of Konstanz, Physical Chemistry, Konstanz 78457, Germany; Max Planck Institute for Chemical Energy Conversion, Mülheim an der Ruhr 45470, Germany; Max Planck Institute for Chemical Energy Conversion, Mülheim an der Ruhr 45470, Germany; School of Materials Science and Engineering, Ocean University of China, Qingdao 266100, China; Qingdao New Energy Shandong Laboratory, Qingdao Institute of Bioenergy and Bioprocess Technology, Chinese Academy of Sciences, Qingdao 266101, China; University of Konstanz, Physical Chemistry, Konstanz 78457, Germany

**Keywords:** metal hydroxide, formation mechanism, *in situ* characterization, coordination geometry, oxygen evolution reaction

## Abstract

Transition metal hydroxides, comprising metal-centered polyhedra, are abundant in nature and have a broad range of applications. Although the intercalation/deintercalation of polyhedra with unconventional coordination numbers (UCN) plays a pivotal role in their formation process and influences their chemical behavior, the detailed mechanism remains obscure. Here, taking the kinetically controlled growth of Co(OH)_2_ as a model system (where polyhedra with UCN refers to 4-coordinated tetrahedra), by combining *in situ* pH measurement and *in situ* ultraviolet–visible (UV–vis) spectroscopy, we tracked Co^2+^ tetrahedral intercalation/deintercalation with varying OH^−^ concentrations, discovering that the initial Co^2+^ tetrahedral intercalation into the Co(OH)_2_ lattice is inevitable and its retention is influenced by the effective OH^−^ concentration, challenging previous beliefs about the formation of Co(OH)_2_ and potentially other hydroxides. More importantly, an understanding of the intercalation mechanism would significantly contribute to optimization of the synthesis conditions of hydroxides with tunable coordination environments, which hold significant application potential, as evidenced by a proof-of-concept application in the oxygen evolution reaction.

## Teaser

This study reveals how reaction conditions shape the coordination environment of Co sites within the cobalt hydroxide structure, advancing the development of adaptable materials for some critical applications.

## INTRODUCTION

Transition metal hydroxides (TMHs) not only are ubiquitous in the environment and provide a model system for the nucleation and crystallization analysis of hydroxide-rich minerals [1–3], but also exhibit captivating and promising properties across various applications [4–11]. Wet chemical synthesis is the most widely used preparation method for TMHs [12–16], which involves a series of transformation processes of water/anion-coordinated metal ions driven by an increase in the OH^−^ concentration [17,18]. This leads to the formation of an insoluble network composed of multiple anions that coordinate with the metal-centered polyhedra, which can appear as either exclusive polyhedra with conventional coordination numbers (such as for bivalent TMHs such as Fe, Co, Ni, Mn, Cu and Zn, where the conventional coordination number is 6) or a coexistence of polyhedra with both conventional and unconventional coordination numbers (UCN) [19]. The intercalation/deintercalation of polyhedra with UCN plays a vital role in the nucleation, growth, composition, structure, size and morphology of TMHs, thus exerting great influence on both fundamental research and practical applications of TMHs [6,20,21]. To name a few, the existence of additional 4-coordinated polyhedra is commonly used to explain how certain TMHs with intercalated guest anions maintain charge neutrality [22]. Additionally, the incorporation of unconventional non-6-coordinated polyhedral metal complexes emerges as an effective strategy for modulating the coordination environment of TMHs, thereby enhancing their catalytic performance [23]. Furthermore, recent investigations suggest a profound correlation between switchable metal (NiO_6_ and NiO_4_) and oxygen redox chemistry in nickel-oxyhydroxide-based materials, triggered by light [24]. Despite the broad interest and value, previous studies have largely focused on the improvement of synthesis methods and the performance of TMHs for target applications through an empirical ‘trial-and-error’ approach and limited attention has been directed towards investigating the dynamic intercalation/deintercalation process of polyhedra with UCN. In addition to this lack of focused efforts, *ex situ* techniques fall short in providing insights into the intercalation mechanism due to the information deviation between the formed intermediate phases during the reaction and the final static TMHs. Nevertheless, the implementation of *in situ* techniques, which is crucial for real-time insights into reaction kinetics, faces substantial challenges, as outlined below. Firstly, finding affordable and accessible *in situ* techniques for monitoring the transient intercalation/deintercalation of ultra-small polyhedra is challenging. Secondly, understanding the intercalation mechanism requires simultaneous determination of the OH^−^ concentration and dynamic intercalation/deintercalation behavior, adding complexity to *in situ* studies. Thirdly, harsh reaction conditions and interrelated parameters (including temperature, solvent, pressure, additives, etc.) in current synthesis strategies limit the *in situ* characterization of TMH formation feasibility. The aforementioned circumstances have led to sluggish progress in understanding the intercalation mechanism of polyhedra with UCN. As a result, we are unable to achieve controlled modulation of the coordination environment of metal sites and are struggling to fully comprehend some mechanistic questions such as why the same TMH has different coordination environments under varying preparation conditions. It is therefore both necessary and urgent to investigate the intercalation mechanism of polyhedra with UCN with the help of a deliberately designed combination of *in situ* techniques and synthesis methods.

Here, we suggest using a combined multimodal characterization method, which involves *in situ* pH measurement and *in situ* ultraviolet–visible (UV–vis) spectroscopy, to monitor the evolving behavior of polyhedra with UCN during the precipitation process of Co(OH)_2_ induced by the kinetically controlled introduction of NaOH or NH_3_. The ingenuity of this experimental design is highlighted by: (i) for Co(OH)_2_, a polyhedron with UCN adopts a tetrahedral coordination geometry that is UV–vis-active [25,26], allowing direct observation of its intercalation/deintercalation through real-time UV–vis signal analysis; (ii) the combination of *in situ* pH and *in situ* UV–vis measurements can synchronously and simultaneously determine OH^−^ concentration and the dynamic behavior of the Co^2+^ tetrahedron; (iii) kinetic control of the base introduction slows the reaction rate, allowing the correlation between tetrahedral Co^2+^ intercalation/deintercalation and real-time OH^−^ concentration changes; (iv) the use of NaOH and NH_3_, which are common strong and weak bases for TMHs synthesis, allows comprehensive exploration.

## RESULTS

**Figure 1.**
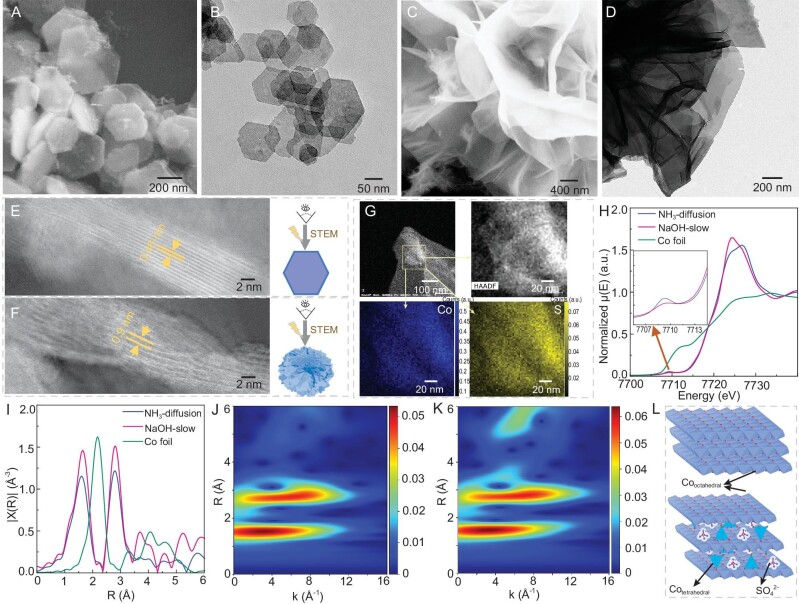
SEM and TEM images of (A) and (B) NaOH-slow and (C) and (D) NH_3_-diffusion. STEM images of (E) NaOH-slow and (F) NH_3_-diffusion viewed from the direction parallel to the lateral surface, along with schematic diagrams of such a characterization method. (G) STEM element mapping images of NH_3_-diffusion. (H) Normalized Co K-edge XANES spectra. (I) Fourier-transformed k^2^-weighted R-space EXAFS spectra. Wavelet-transformed EXAFS plots of (J) NH_3_-diffusion and (K) NaOH-slow. (L) Schematic diagrams of the structures of NaOH-slow and NH_3_-diffusion.

Specifically, NaOH solution (2.5 mL, 0.8 M) and NH_3_ from 2 mL commercial NH_3_·H_2_O were introduced into an aqueous solution of CoSO_4_·7H_2_O at room temperature without using any additives via program-controlled dropwise addition and continuous gas diffusion, respectively (Fig. S1). The resulting products were labeled as NaOH-slow and NH_3_-diffusion, characterized by hexagonal nanosheet morphology (with sizes ranging from 50 to 200 nm) and a flower-like structure with numerous nanosheet units, respectively, which were evident from scanning electron microscopy (SEM) and transmission electron microscopy (TEM) images (Fig. 1A–D). The powder X-ray diffraction (XRD) pattern of NaOH-slow (Fig. S2A) shows characteristic peaks for β-Co(OH)_2_ without intercalated anions [27,28]. Although the XRD pattern of NH_3_-diffusion (Fig. S2B) cannot be directly indexed to the standard powder diffraction file, it is reasonable to classify NH_3_-diffusion as α-Co(OH)_2_ due to its similarity to the XRD pattern reported in previous studies [29,30]. Three diffraction peaks at 2θ values of 8.1°, 16.3° and 33.2° are in agreement with (003), (006) and (009) planes of a hydrotalcite-like structure, respectively [31]. Compared with the (001) peak in the XRD pattern of NaOH-slow, the (003) peak in the XRD pattern of NH_3_-diffusion exhibits a shift towards lower angles, indicating increased interlayer spacing. This difference in interlayer spacing between the two samples is further confirmed by using scanning transmission electron microscopy (STEM) to analyse the direction parallel to the lateral surface. As shown in Fig. 1E and F, the neighboring layers of NaOH-slow present an interplanar spacing of 0.46 nm whereas the interplanar spacing of NH_3_-diffusion is 0.9 nm. Notably, the increased interplanar spacing of NH_3_-diffusion can be attributed to the intercalation of SO_4_^2−^ into the interlayers. This is supported by Fourier transform infrared (FTIR) spectroscopy results (Fig. S3), which show the presence of SO_4_^2−^ in the NH_3_-diffusion system and its absence in the NaOH-slow system. Additionally, multivariate statistical analysis [32] of energy dispersive X-ray (EDX) spectrum imaging (Fig. S4) reveals S in the NH_3_-diffusion system whereas it is not detected in the NaOH-slow system. Furthermore, the distribution of S in the NH_3_-diffusion system is highly homogeneous (Fig. 1G).

X-ray absorption spectroscopy (XAS) studies were conducted to investigate the local chemical environment of the Co sites. Figure 1H–K shows the Co K-edge X-ray absorption near edge spectroscopy (XANES) spectra and the corresponding extended X-ray absorption fine structure (EXAFS) spectra. At the pre-edge region of the XANES spectra, compared with NaOH-slow, NH_3_-diffusion presents a more pronounced pre-edge feature at 7709.2 eV, which corresponds to transitions from the 1s core level to the unoccupied 3d orbitals. The increase in the intensity of the pre-edge feature could be contributed from a fraction of the tetrahedral Co^2+^ sites in the NH_3_-diffusion because, compared with the 6-coordinated octahedral geometry, the 4-coordinated tetrahedral geometry is a less centrosymmetric configuration with stronger d–p hybridization between the Co site and the surrounding O/OH ligands, which leads to more p-character in the 3d orbitals, more dipole-allowed character in the 1s–3d transitions and correspondingly increased pre-edge intensities. Meanwhile, the EXAFS spectra of these two samples also suggest differences in coordination structure (Fig. 1I–K, Fig. S5 and Table S1). First, the coordination number (CN) of Co–O is 5.2 ± 0.7 for NH_3_-diffusion, which is less than that of NaOH-slow (5.9 ± 0.6), indicating the coexistence of 6-coordinated and 4-coordinated Co sites in the structure. Second, both samples have very similar CNs for the second shell Co–Co coordination but the Debye–Waller factors of both the Co–O path and the Co–Co path are relatively larger for NH_3_-diffusion. This is also in agreement with the presence of 4-coordinated tetrahedral Co sites, as the coordination environment of Co sites is less uniform compared with that in NaOH-slow. Last but not least, as shown in Fig. 1I–K, the EXAFS of NH_3_-diffusion shows much less pronounced scattering features at >3.5 Å. When considering the structure of α-Co(OH)_2_ compared with that of β-Co(OH)_2_ (Table S2), the most significant difference is that α-Co(OH)_2_ has intercalated guest anions that make the interatomic distances between the atoms from different layers become significantly elongated. Therefore, several Co–O(H) and Co–Co scattering paths at >3.5 Å only exist in the β-Co(OH)_2_ structure but not in the α-Co(OH)_2_ structure. On top of this fact, it is concluded that NH_3_-diffusion exhibits a coexistence of both 4-coordinated tetrahedral and 6-coordinated octahedral Co sites, resembling the structure of α-Co(OH)_2_ whereas NaOH-slow is more similar to β-Co(OH)_2_, featuring exclusive octahedral Co sites (see schematic diagrams of their structures in Fig. 1L). The presence or absence of tetrahedral Co^2+^ can also be determined by whether there are two absorption peaks between 550 and 650 nm in the UV–vis absorption spectrum based on the fact that d–d transitions are permitted for tetrahedral Co^2+^ (d^7^) due to the lack of a symmetry center while d–d transitions are forbidden in octahedral Co^2+^ [23]. The UV–vis absorption spectrum of NH_3_-diffusion reveals the existence of two absorption peaks between 550 and 650 nm whereas these two absorption peaks are not observed in the UV–vis absorption spectrum of NaOH-slow (Fig. S6), indicating the existence of tetrahedral Co^2+^ in NH_3_-diffusion and its absence in NaOH-slow.

### *In situ* pH and UV–vis studies of the intercalation/deintercalation of tetrahedral Co^2+^

Based on the aforementioned information, monitoring of the dynamic intercalation/deintercalation process of tetrahedral Co^2+^ can be effectively accomplished through *in situ* UV–vis measurements, eliminating the need for XAS techniques that are both limited in availability and associated with high costs. Besides, the kinetically controlled addition of NaOH or NH_3_ serves to decelerate the reaction rate, facilitating the observation of variations in OH^−^ concentrations throughout the reaction. This is achieved by recording the real-time pH by using an ion-selective electrode tailored for H^+^ ions. Consequently, the combination of *in situ* pH and *in situ* UV–vis spectroscopy to characterize the reaction processes of NH_3_-diffusion and NaOH-slow presents a promising model system for investigating the intercalation/deintercalation mechanism of tetrahedral Co^2+^.

**Figure 2.**
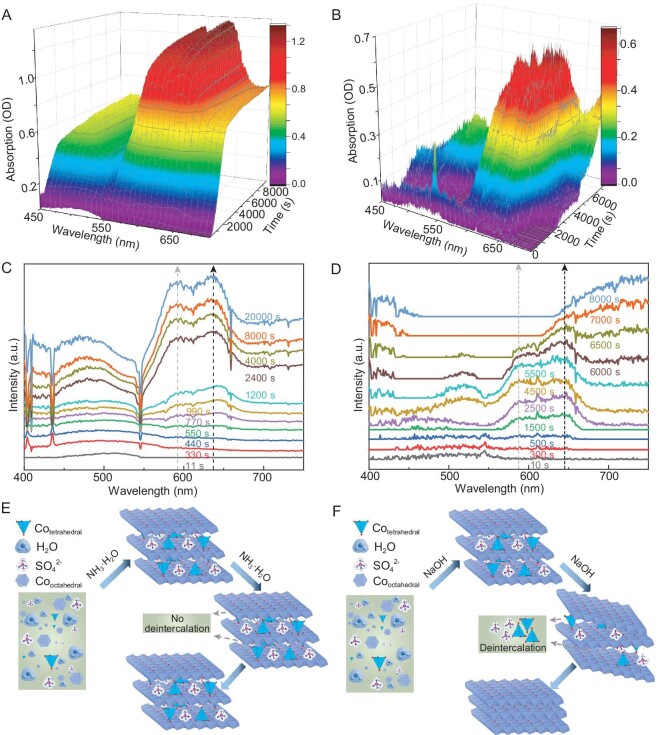
*In situ* UV–vis spectra as a function of immersion time for the reaction process of (A) NH_3_-diffusion and (B) NaOH-slow. UV–vis spectra at representative time points for the reaction process of (C) NH_3_-diffusion and (D) NaOH-slow. Illustration of the intercalation and deintercalation of tetrahedral Co^2+^ during the reaction process of (E) NH_3_-diffusion and (F) NaOH-slow.

Accordingly, the reaction process of NH_3_-diffusion and NaOH-slow was characterized by *in situ* pH and *in situ* UV–vis measurements. In Fig. S7A, the pH profile of NH_3_-diffusion unfolds in distinct stages. Initially, a pH increase correlates with the gradual introduction of NH_3_, resulting in a rising OH^−^ concentration. Subsequently, the pH curves reach a constant plateau, indicating equilibrium between OH^−^ introduction and OH^−^ consumption through the hydrolysis of Co^2+^ ions. Before reaching the final plateau, there is a swift pH ascent, signaling a higher OH^−^ generation rate than its consumption rate, signaling the near-completion of Co^2+^ ion hydrolysis. The *in situ* UV–vis results that are shown in Fig. 2A and C reveal two absorption peaks at ∼590 and ∼630 nm during NH_3_-diffusion. These peaks, corresponding to tetrahedral Co^2+^, emerge concurrently with the onset of the first pH plateau and subsequently increase in intensity, eventually stabilizing with ongoing OH^−^ introduction. Based on this, we conclude that the dynamic evolution process of tetrahedral Co^2+^ during the NH_3_-diffusion reaction is as follows: as shown in Fig. 2E, with the progression of the Co^2+^ hydrolysis process induced by OH^−^ introduction, tetrahedral Co^2+^ is intercalated into the hydroxide lattice and, as the reaction proceeds, the initially intercalated tetrahedral Co^2+^ is successfully retained in the lattice without deintercalation. While the pH profile of NaOH-slow is similar to that of NH_3_-diffusion (Fig. S7B), the evolution of the two absorption peaks at 590 and 630 nm in the *in situ* UV–vis spectra reveals distinct behaviors (Fig. 2B and D). Specifically, these two absorption peaks appeared at the onset time of the first pH plateau and, as the reaction progressed, their intensities exhibited the following trend: a gradual increase, reaching a stable level, followed by a subsequent gradual decrease until they disappeared. This indicates the intercalation and subsequent deintercalation of tetrahedral Co^2+^ during the reaction process of NaOH-slow, as illustrated in Fig. 2F. Notably, the significant difference in the band intensity of the two UV–vis absorption peaks between Fig. 2A and B indicates that the concentration of Co^2+^ tetrahedral in the two products is markedly different, highlighting the substantial differences in their formation processes.

**Figure 3.**
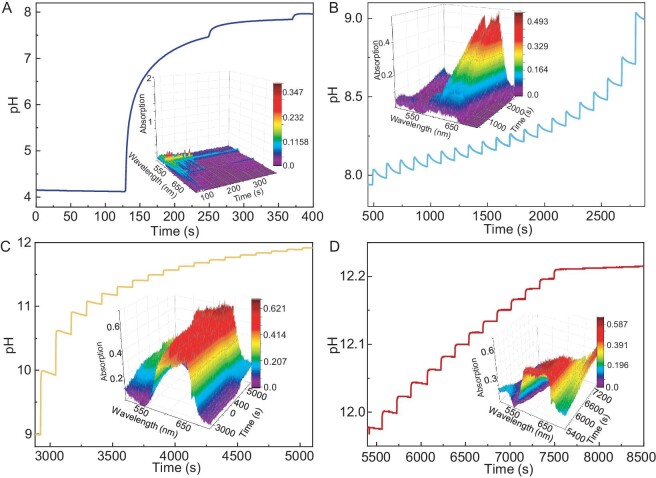
The pH profiles and UV–vis spectra at different reaction stages for the reaction process of NaOH-slow. The pH profiles exhibit step-like patterns due to the dropwise addition of NaOH solution (one drop every 120–180 seconds, with each drop being 40–60 µL).

To comprehensively understand the correlation between pH variations and the intercalation/deintercalation of tetrahedral Co^2+^ during the reaction process of NaOH-slow, a detailed analysis of *in situ* UV–vis spectra and *in situ* pH profiles was conducted. It was discovered that, in the initial stage (Fig. 3A), each addition of NaOH solution causes a rapid increase in pH, and the absence of the two absorption peaks at 590 and 630 nm indicates that this stage precedes the intercalation of tetrahedral Co^2+^. In the second stage (Fig. 3B), the addition of NaOH solution each time leads to an initial increase and subsequent decrease in pH, with the decrease in pH attributed to the hydrolysis of Co^2+^ ions consuming OH^−^. Meanwhile, the two absorption peaks begin to appear and their intensities gradually increase, indicating the onset of Co^2+^ hydrolysis and the initiation of the intercalation of tetrahedral Co^2+^. In the third stage (Fig. 3C), the pH trend is similar to that in the second stage but with a slower rate of decrease compared with the previous stage. Simultaneously, the intensity of those two absorption peaks gradually increases and reaches a stable stage, probably due to the gradually decreased hydrolysis rate of Co^2+^. In the final stage, the hydrolysis process of Co^2+^ ions is nearing completion and each addition of NaOH solution causes a rapid increase in pH, which then stabilizes until the next addition of NaOH (Fig. 3D). Meanwhile, the intensities of the two absorption peaks gradually decrease and eventually disappear, indicating that excessive NaOH leads to the deintercalation of tetrahedral Co^2+^. The above results reveal that the intercalation of tetrahedral Co^2+^ is a commonly occurring phenomenon irrespective of whether NaOH or NH_3_ is used as the base source, and the retention of initially intercalated tetrahedral Co^2+^ hinges on the specific reaction conditions. The difference between these two reaction processes of NaOH-slow and NH_3_-diffusion lies in the change rate of OH^−^, the amount of OH^−^ and the presence/absence of NH_4_^+^ in the reaction solution. Considering these factors, alongside the successful retention of tetrahedral Co^2+^ in NH_3_-diffusion and the total deintercalation of tetrahedral Co^2+^ in NaOH-slow, an investigation into the influence of these reaction parameters on the retention of initially intercalated tetrahedral Co^2+^ becomes imperative.

### Identifying factors influencing the intercalation/deintercalation of tetrahedral Co^2+^

We initially investigated whether the reaction rate plays a pivotal role in determining the successful retention of tetrahedral Co^2+^ in the final product. Two additional experiments with fast reaction rates were carried out via pouring commercial NH_3_·H_2_O (2 mL) and NaOH solution (2.5 mL, 0.8 M) into the reaction solution of CoSO_4_·7H_2_O. The obtained products were denoted as NH_3_·H_2_O-pour and NaOH-pour-4.55:1-CoSO_4_·7H_2_O (where #:# represents the molar ratio of OH^−^ to Co^2+^), respectively, and the reaction processes were monitored by using *in situ* pH and *in situ* UV–vis measurements. As depicted in Fig. S8A and B, the pH immediately rises to ∼12 upon the addition of the base source, attributed to the rapid release of OH^−^. Regarding the two absorption peaks observed in the *in situ* UV–vis spectra, their changing patterns in NH_3_·H_2_O-pour (Fig. S9A and C) resemble those observed in NH_3_-diffusion, while the evolving patterns in NaOH-pour-4.55:1-CoSO_4_·7H_2_O (Fig. S9B and D) display similarities to those observed in NaOH-slow. This indicates that tetrahedral Co^2+^ is initially incorporated and finally retained in NH_3_·H_2_O-pour, while NaOH-pour-4.55:1-CoSO_4_·7H_2_O undergoes the process of tetrahedral Co^2+^ intercalation followed by subsequent deintercalation. The presence or absence of tetrahedral Co^2+^ was indirectly confirmed by XRD results as well. Specifically, the XRD pattern of NaOH-pour-4.55:1-CoSO_4_·7H_2_O (Fig. S10) exhibits characteristic peaks for β-Co(OH)_2_ without intercalated guest anions and the XRD pattern of NH_3_·H_2_O-pour is similar to that of NH_3_-diffusion that has been confirmed to be α-Co(OH)_2_ with the intercalated guest anions. The intercalation of guest anions serves as indirect evidence of the simultaneous intercalation of tetrahedral Co^2+^, as previous studies have established that both processes occur simultaneously to maintain charge neutrality [25,26]. Based on the aforementioned analysis, we conclude that an alteration in the reaction rate has minimal impact on the intercalation and deintercalation behavior of tetrahedral Co^2+^. Note that the pH profiles of NH_3_·H_2_O-pour and NaOH-pour-4.55:1-CoSO_4_·7H_2_O provide limited useful information regarding the time-dependent pH changes, making it challenging to establish a correlation between the intercalation/deintercalation of tetrahedral Co^2+^ and real-time variations in OH^−^ concentrations. This underscores the strategic choice to employ the reaction processes of NH_3_-diffusion and NaOH-slow, with kinetically controlled reaction rates, as model systems for investigating the intercalation/deintercalation mechanism.

**Figure 4.**
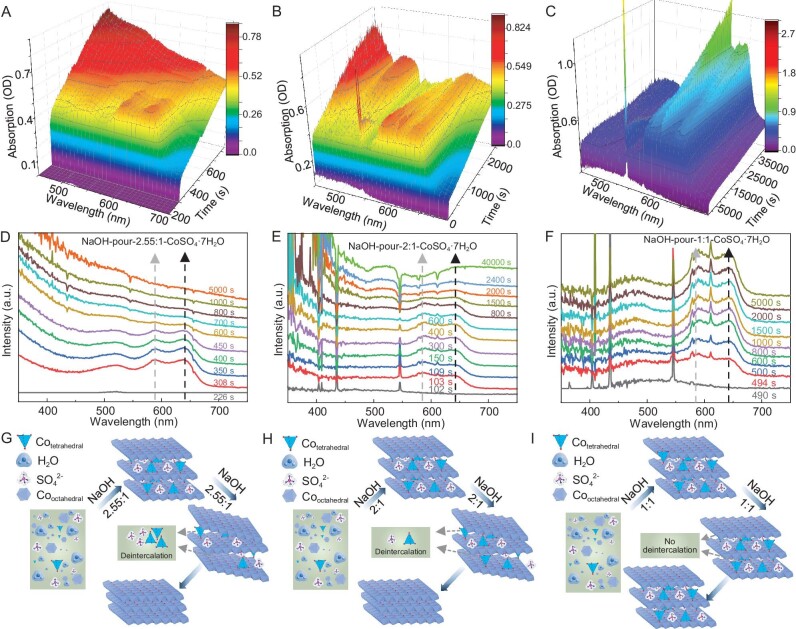
*In situ* UV–vis spectra as a function of immersion time for the reaction process of (A) NaOH-pour-2.55:1-CoSO_4_·7H_2_O, (B) NaOH-pour-2:1-CoSO_4_·7H_2_O and (C) NaOH-pour-1:1-CoSO_4_·7H_2_O. UV–vis spectra at representative time points for the reaction process of (D) NaOH-pour-2.55:1-CoSO_4_·7H_2_O, (E) NaOH-pour-2:1-CoSO_4_·7H_2_O and (F) NaOH-pour-1:1-CoSO_4_·7H_2_O. Illustration of the intercalation and deintercalation of tetrahedral Co^2+^ during the reaction process of (G) NaOH-pour-2.55:1-CoSO_4_·7H_2_O, (H) NaOH-pour-2:1-CoSO_4_·7H_2_O and (I) NaOH-pour-1:1-CoSO_4_·7H_2_O.

**Figure 5.**
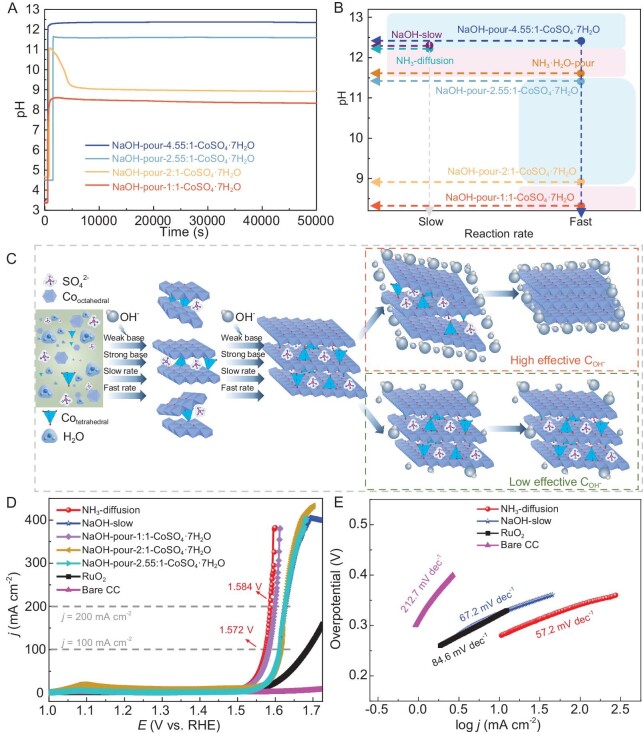
(A) Observation of pH as a function of time for the reaction process of NaOH-pour-4.55:1-CoSO_4_·7H_2_O, NaOH-pour-2.55:1-CoSO_4_·7H_2_O, NaOH-pour-2:1-CoSO_4_·7H_2_O and NaOH-pour-1:1-CoSO_4_·7H_2_O. (B) Relationship between pH of the final reaction solution, reaction rate and the deintercalation/retention of tetrahedral Co^2+^. The regions shaded in blue and pink highlight the deintercalation and retention of tetrahedral Co^2+^, respectively. (C) Illustration of the proposed intercalation/deintercalation mechanism. (D) LSV curves and (E) Tafel slopes of different electrocatalysts.

The relationship between the intercalation/deintercalation of tetrahedral Co^2+^ and the added amount of OH^−^ is also worth validating through conducting control experiments. Here, NaOH is used as the base source to eliminate the influence of NH_4_^+^. The NaOH solution can be added to the reaction solution by program-controlled dropwise addition or pouring when conducting the contrast experiments. The method of pouring NaOH solution into the reaction solution was chosen instead of the program-controlled dropwise addition of NaOH solution because the latter method could result in inconsistencies in the time of adding NaOH solutions with different volumes, which becomes a potential confounding factor that could affect the reaction process. Therefore, a series of control experiments were carried out under the same reaction conditions as NaOH-pour-4.55:1-CoSO_4_·7H_2_O, with the only difference being that the volume of NaOH solution was changed to 0.55, 1.1 and 1.4 mL. The amount of OH^−^ present in 1.1 mL of NaOH solution is in a 2:1 molar ratio to the Co^2+^ ions in the solution. The resulting products were denoted as NaOH-pour-1:1-CoSO_4_·7H_2_O, NaOH-pour-2:1-CoSO_4_·7H_2_O and NaOH-pour-2.55:1-CoSO_4_·7H_2_O, respectively. On the one hand, as shown in Fig. 4A, B, D and E, the changing trend of the two absorption peaks at 590 and 630 nm in the *in situ* UV–vis spectra of NaOH-pour-2.55:1-CoSO_4_·7H_2_O and NaOH-pour-2:1-CoSO_4_·7H_2_O exhibits similarity to that of NaOH-pour-4.55:1-CoSO_4_·7H_2_O—namely, an initial increase, followed by a short period of stability and then a decrease until they disappear. This indicates the initial intercalation and subsequent deintercalation of tetrahedral Co^2+^, as illustrated in Fig. 4G and H. The deintercalation of tetrahedral Co^2+^ in the resulting products is indirectly supported by the absence of intercalated SO_4_^2−^ anions. This is evidenced by the lack of a shift in the (003) peak to smaller angles in the XRD results (Fig. S11A and B), the absence of a peak corresponding to SO_4_^2–^ in the FTIR results (Fig. S12) and the lack of an S signal in the EDX spectrum (Fig. S13). The deintercalation rate of tetrahedral Co^2+^ is related to the added amount of NaOH solution, which is supported by the decreasing duration of those two absorption peaks as the added amount of NaOH increases (∼2300 s for NaOH-pour-2:1-CoSO_4_·7H_2_O, ∼500 s for NaOH-pour-2.55:1-CoSO_4_·7H_2_O and ∼105 s for NaOH-pour-4.55:1-CoSO_4_·7H_2_O). In contrast, the two absorption peaks at 590 and 630 nm for NaOH-pour-1:1-CoSO_4_·7H_2_O were initially observed to increase and subsequently remain constant (Fig. 4C and F), suggesting the successful intercalation and retention of tetrahedral Co^2+^ (Fig. 4I). The presence of a shifted (003) peak towards lower angles in the XRD result of NaOH-pour-1:1-CoSO_4_·7H_2_O (Fig. S14) confirms the successful intercalation of SO_4_^2−^ into Co(OH)_2_. Additionally, the successful intercalation of SO_4_^2–^ is further supported by the following evidence: the presence of a peak corresponding to SO_4_^2–^ in the FTIR results (Fig. S12) and the detection of an S signal in the EDX spectrum (Fig. S13). Moreover, the successful intercalation of SO_4_^2–^ can be considered to be indirect evidence supporting the retention of tetrahedral Co^2+^ in NaOH-pour-1:1-CoSO_4_·7H_2_O. To gain more insight into whether the initial intercalation and subsequent deintercalation of 4-coordinated Co affects the morphology of hydroxides, we used SEM to investigate the morphology of the hydroxides obtained by adding different amounts of NaOH. As shown in Fig. S15A, with a smaller amount of NaOH, the resulting product exhibits an irregular sheet-like morphology. However, when the amount of NaOH is increased to a certain level (Fig. S15B–D), the morphology of the hydroxides changes to a hexagonal shape. This indicates that the initial intercalation and subsequent deintercalation of 4-coordinated Co promote the morphological transformation of hydroxides. On the other hand, the pH profiles of NaOH-pour-1:1-CoSO_4_·7H_2_O, NaOH-pour-2:1-CoSO_4_·7H_2_O and NaOH-pour-2.55:1-CoSO_4_·7H_2_O are illustrated in Fig. 5A, with the inclusion of the pH profile of NaOH-pour-4.55:1-CoSO_4_·7H_2_O for comparative analysis. It can be seen that, at the moment of adding the NaOH solution, there is an instantaneous increase in pH, followed by a pH decrease process before reaching a pH plateau. The plateau pH decreases with a reduction in the initial amount of added NaOH, indicating that the concentration of OH^−^ in the final reaction solution is different. Based on the aforementioned discussions, it is evident that the differentiating factor among the reaction processes of NaOH-pour-1:1-CoSO_4_·7H_2_O, NaOH-pour-2:1-CoSO_4_·7H_2_O, NaOH-pour-2.55:1-CoSO_4_·7H_2_O and NaOH-pour-4.55:1-CoSO_4_·7H_2_O lies solely in the OH^−^ concentration in the reaction solution. Simultaneously, variations in the intercalation/deintercalation behavior of tetrahedral Co^2+^ are observed during these reaction processes, where deintercalation becomes more facile and rapid with an increase in the OH^−^ concentration; conversely, reducing the OH^−^ concentration to a certain level facilitates the successful retention of tetrahedral Co^2+^. These discoveries lead us to speculate that OH^−^ in the solution tends to competitively induce the deintercalation of the initially intercalated tetrahedral Co^2+^ and the competitiveness of OH^−^ is related to its concentration.

Although the aforementioned speculation provides a solid understanding of the deintercalation/retention of tetrahedral Co^2+^ during the reaction process when using NaOH as the base source, it fails to adequately explain why tetrahedral Co^2+^ can be retained in NH_3_·H_2_O-pour while it is deintercalated in NaOH-pour-2.55:1-CoSO_4_·7H_2_O, despite both NH_3_·H_2_O-pour and NaOH-pour-2.55:1-CoSO_4_·7H_2_O possessing similar final plateau pH levels (Fig. 5B). Similarly, the same question arises in the comparison between NH_3_-diffusion and NaOH-slow. These results suggest that the intercalation/deintercalation of tetrahedral Co^2+^ is undoubtedly influenced by the presence of NH_3_. Theoretically, NH_3_ could affect Co(OH)_2_ formation in two ways: first, by forming complexes with Co^2+^ and, second, by interacting with H_2_O to form NH_3_·H_2_O, which, compared with NaOH, is a weaker base with a lower ability to release OH^−^. To investigate whether NH_3_ forms complexes with Co^2+^ and thereby influences Co(OH)_2_ formation in our system, we collected new UV–vis spectra with an improved signal-to-noise ratio by periodically extracting samples from the reaction solution during both the NH_3_ diffusion and NaOH-slow processes, leveraging UV–vis spectroscopy as a powerful tool for detecting [Co(NH_3_)_6_]^2+^ complexes [33]. In the quasi-*in situ* UV–vis spectra for the NaOH-slow reaction (Fig. S16A), we observed an initial peak at 500 nm before the introduction of NaOH, corresponding to the [Co(H_2_O)_6_]^2+^ complex [33]. As NaOH was gradually added, the peak initially shifted to ∼470 nm and then gradually shifted back as the reaction progressed. Interestingly, the shift back of the peak from 470 nm coincides with the decrease in the intensity of the peaks at ∼600 nm, which represents the presence of 4-coordinated Co tetrahedra. This leads us to deduce that the shift in the peak position is strongly related to the intercalation/deintercalation of 4-coordinated tetrahedra in cobalt hydroxide. For the NH_3_-diffusion reaction, the quasi-*in situ* UV–vis spectra (Fig. S16B) also showed a peak at 500 nm that corresponded to the [Co(H_2_O)_6_]^2+^ complex before the introduction of NH_3_. As NH_3_ was gradually introduced, the peak shifted to ∼470 nm and stabilized, and this occured simultaneously with the appearance and subsequent stabilization of the peaks at ∼600 nm. Interestingly, after the introduction of NH_3_, the 500-nm peak characteristics of both [Co(H_2_O)_6_]^2+^ and [Co(NH_3_)_6_]^2+^ disappeared [33]. This suggests that [Co(NH_3_)_6_]^2+^ complexes either did not form or were present in extremely low concentrations. Additionally, the trend observed in the quasi-*in situ* UV–vis spectra for the NH_3_-diffusion system closely resembles that of the NaOH-pour-1:1-CoSO₄·7H₂O reaction (Fig. S16C). As NH_3_ is absent in the NaOH-pour-1:1-CoSO_4_·7H_2_O system, this similarity further confirms that the reactions in the NH_3_-diffusion and NaOH-pour-1:1-CoSO_4_·7H_2_O systems are remarkably similar. This supports the conclusion that [Co(NH_3_)_6_]^2+^ does not form or plays no significant role in the reaction process. This is also consistent with the absence of N in the final product of NH_3_-diffusion, which is confirmed by the lack of N–H peaks in the FTIR spectrum (Fig. S3) of NH_3_-diffusion and the absence of an N signal in the EDX spectrum (Fig. S4). With the role of NH_3_ in forming [Co(NH_3_)_6_]^2+^ complexes having been ruled out, the only remaining explanation for its effect on the intercalation/deintercalation of tetrahedral Co^2+^ is the weak releasing OH^−^ ability of NH_3_·H_2_O compared with NaOH. NaOH and NH_3_ are the most commonly used strong base and weak base, respectively, for preparing hydroxides and they have different dissociation favorabilities to generate OH^−^. To be specific, by comparing the magnitudes of the Gibbs energy (–∆_*r*_*G*°_*m*_(*T*)) values of the following two equations [13], we can conclude that Equation (1) is non-spontaneous and less favorable whereas Equation (2) is spontaneous and favorable under standard conditions:


(1) \begin{eqnarray*}
{\mathrm{N}}{{\mathrm{H}}}_3\! \cdot\! {{\mathrm{H}}}_2{\mathrm{O}}\! \rightleftharpoons\! {\mathrm{N}}{{\mathrm{H}}}_4^ + \! +\! {\mathrm{O}}{{\mathrm{H}}}^ - , - {\Delta }_rG_m^{\circ}(T)\! =\! 271\,{\mathrm{kJ}},
\end{eqnarray*}

(2) \begin{eqnarray*}
{\mathrm{NaOH}} \to {\mathrm{N}}{{\mathrm{a}}}^ + + {\mathrm{OH}}^{-} , - {\Delta }_r{G}^{\circ}_m(T) = - 44\,{\mathrm{kJ}}.
\end{eqnarray*}

In this case, acquiring a free OH^−^ that can be consumed by the hydrolysis of Co^2+^ is more difficult when using NH_3_ as the base source compared with obtaining a free OH^−^ from NaOH solution. This is further supported by the fact that, compared with the case of NaOH-slow, the pH plateau appears at higher OH^−^ concentrations under NH_3_-diffusion conditions (Fig. S7A and B), at approximately C_OH_^−^ = 10^−6^ mol/L (pH = 8, NaOH-slow) and C_OH_^−^ = 2.51 × 10^−6^ mol/L (pH = 8.4, NH_3_-diffusion). This implies that, to achieve the same effective OH^−^ concentration that is required to facilitate the hydrolysis of Co^2+^, a higher actual concentration of OH^−^ is needed in NH_3_-diffusion compared with NaOH-slow. To further validate the impact of the presence of NH_3_/NH_4_^+^ on the concentration of effective OH^−^, two additional experiments were carried out. The first was performed under the same reaction conditions as NaOH-pour-2.55:1-CoSO_4_·7H_2_O except that aqueous solution containing CoSO_4_·7H_2_O was replaced by aqueous solution containing CoSO_4_·7H_2_O and NH_4_Cl (0.07 M). The purpose of adding NH_4_Cl was to verify whether the introduction of NH_4_^+^ ions, which can induce the aforementioned reversible Equation (1), could reduce the effective OH^−^ concentration in the reaction system of NaOH-pour-2.55:1-CoSO_4_·7H_2_O and prevent the deintercalation of tetrahedral Co^2+^. The obtained product was denoted as NaOH-pour-2.55:1-CoSO_4_·7H_2_O-NH_4_Cl. Its XRD pattern, as shown in Fig. S17A, shows a shifted (003) peak towards lower angles, which suggests the successful intercalation of SO_4_^2−^, indirectly indicating the presence of tetrahedral Co^2+^. Thus, to some extent, the deintercalation of tetrahedral Co^2+^ is partially inhibited, confirming that the presence of NH_4_^+^ indeed reduces effective OH^−^ concentration in the solution. The second additional experiment involves a pair of control experiments, A and B. In Experiment A, the reaction solution containing CoSO_4_·7H_2_O was placed in a sealed desiccator with 2 mL of commercial NH_3_·H_2_O for 12 hours of NH_3_ diffusion. Subsequently, 1.4 mL of 0.8 M NaOH solution was added to the reaction solution, followed by a further 12-hour incubation at room temperature. The resulting product was collected and labeled as NH_3_-diffusion-NaOH-pour-2.55:1-CoSO_4_·7H_2_O. Experiment B was similar to Experiment A with the only difference being the removal of NH_3_/NH_4_^+^ through centrifugation (three times with water) before the addition of 1.4 mL of 0.8 M NaOH solution. The obtained product was designated as NH_3_-diffusion-clean-NaOH-pour-2.55:1-CoSO_4_·7H_2_O. By assessing whether the (003) peak in their XRD results (Fig. S17B and C) shifted towards lower angles, we concluded that there was retention of tetrahedral Co^2+^ in NH_3_-diffusion-NaOH-pour-2.55:1-CoSO_4_·7H_2_O and deintercalation of tetrahedral Co^2+^ in NH_3_-diffusion-clean-NaOH-pour-2.55:1-CoSO_4_·7H_2_O. This further supports that the presence of NH_3_/NH_4_^+^ can diminish the effective OH^−^ concentration in the reaction solution and then decelerate or inhibit the deintercalation of tetrahedral Co^2+^.

### Proposed intercalation/deintercalation mechanism and a proof-of-concept application

In theory, the incorporation of tetrahedral Co^2+^ into the final lattice depends on two key factors: initial intercalation and subsequent retention. Synthesizing insights from the preceding discussions, the proposed mechanism for the intercalation/deintercalation of tetrahedral Co^2+^ unfolds as follows.

As shown in Fig. 5C, with an increase in the OH^−^ concentration, water/anion-coordinated Co^2+^ ions undergo a series of transformation processes, resulting in the formation of an insoluble hydroxide network. During this reaction, tetrahedral Co^2+^ tends to initially intercalate into the network, regardless of whether a strong or weak base is used and independently of the rate of change in the OH^−^ concentration.

The retention of tetrahedral Co^2+^ in the network is significantly influenced by the effective OH^−^ concentration in the reaction solution. The competitive efficacy of effective OH^−^ is closely tied to its actual concentration, along with other factors such as the presence of reversible reactions involving OH^−^.

These findings can facilitate the controlled regulation of 4- and 6-coordinated environments, thus enabling the preparation of specific hydroxides that are tailored to the requirements of various applications. For instance, numerous studies have demonstrated that the presence of 4-coordinated Co sites in electrocatalysts can enhance oxygen evolution reaction (OER) performance, as 4-coordinated Co sites can facilitate the adsorption of water molecules and the formation of active high-valent cobalt (hydr)oxide intermediates [34]. Consequently, NH_3_-diffusion in this study was applied as the electrocatalyst for the OER in a proof-of-concept application. Meanwhile, the OER performance of Co(OH)_2_ that was prepared by using NaOH as the base, the commercial RuO_2_ electrocatalyst and bare carbon cloth were also evaluated for comparison. Firstly, linear sweep voltammetry (LSV) curves of these catalysts were tested at a scan rate of 5 mV s^−1^ in 1 M KOH solution using a standard three-electrode system. As shown in Fig. 5D, NH_3_-diffusion exhibited an OER overpotential of 277 mV at a current density of 10 mA cm^−2^, which was lower than that of not only the commercial RuO_2_ electrocatalyst, but also most congeneric OER electrocatalysts that are prepared under other relatively harsh synthesis conditions (Table S3), suggesting its superior catalytic activity. Interestingly, NaOH-pour-2.55:1-CoSO_4_·7H_2_O, NaOH-pour-2:1-CoSO_4_·7H_2_O and NaOH-slow showed significantly lower catalytic activity compared with NH_3_-diffusion whereas NaOH-pour-1:1-CoSO_4_·7H_2_O exhibited similar catalytic activity to NH_3_-diffusion. Considering the above discussion in which it was found that 4-coordinated Co was present in NaOH-pour-1:1-CoSO_4_·7H_2_O but absent in the other cobalt hydroxides that were prepared with NaOH, it is evident that the presence of 4-coordinated Co sites can enhance OER performance. Furthermore, as shown in Fig. 5E, NH_3_-diffusion has the lowest Tafel slope (57.2 mV dec^−1^), which indicates more favorable catalytic kinetics and further confirms the excellent catalytic properties of NH_3_-diffusion. Except for the parameters mentioned above, superior long-term stability is also a key concern for OER electrocatalysts. Therefore, the chronopotentiometry curves for NH_3_-diffusion and RuO_2_ were recorded at 10 mA cm^−2^ to compare their long-term stability. As shown in Fig. S18, after 24 hours, the potential of NH_3_-diffusion remained nearly constant whereas commercial RuO_2_ showed a voltage change, signifying the more reliable durability of NH_3_-diffusion compared with commercial RuO_2_ catalysts. Considering the OER performance differences caused by variations in coordination environments, along with the optimized synthesis conditions that were achieved through an understanding of the intercalation mechanism, it is concluded that this study makes a significant contribution to the low-cost, simple and green preparation of hydroxides with tunable coordination environments.

## DISCUSSION

The deliberately designed combination of the kinetically controlled growth of hydroxides, *in situ* pH and UV–vis spectroscopy measurements provides solutions for investigating the intercalation/deintercalation mechanism of polyhedra with UCN. Taking Co(OH)_2_ composed of 6-coordinated octahedron and 4-coordinated tetrahedron as the example, this strategy allows us to monitor real-time changes in OH^−^ concentration and the dynamic intercalation/deintercalation behavior of tetrahedral Co^2+^ synchronously and simultaneously, enabling us to identify the factors that influence the intercalation/deintercalation of tetrahedral Co^2+^ and propose an intercalation/deintercalation mechanism. Regardless of the type of base source used or the rate of OH^−^ concentration change, tetrahedral Co^2+^ tends to be incorporated into the lattice in the early stages of the hydrolysis process of Co^2+^. However, the ultimate retention of tetrahedral Co^2+^ is largely influenced by the effective OH^−^ concentration in the reaction solution and the competitive ability of effective OH^−^ is linked to its concentration, as well as other factors including the presence of reversible reactions involving OH^−^. These findings encourage us to reevaluate the previously held beliefs about the formation processes and mechanisms of Co(OH)_2_ and potentially other hydroxides, while also contributing to the cost-effective synthesis and practical applications of TMHs with tunable coordination environments by optimizing their synthesis conditions, as demonstrated by the application of the obtained materials in OER. Besides, the deliberately designed combinatorial methodology provides a new way in which to explore the formation mechanism of solution-phase growth of TMHs as the OH^−^ concentration changes.

## Supplementary Material

nwae427_Supplemental_File
