# Supplementary material for: Mechanistic insights into the formation of hydroxides with unconventional coordination environments to achieve their cost-effective synthesis
Source: Natl Sci Rev. 2024 Dec 23;12(3):nwae427. doi: 10.1093/nsr/nwae427 (PMC11804801; doi:10.1093/nsr/nwae427)
Supplement: nwae427_Supplemental_File [file nwae427_supplemental_file.pdf]

Mechanistic Insights into the Formation of Hydroxides with Unconventional Coordination Environments to Achieve Their Cost-Effective Synthesis

Materials

$CoSO_4 \cdot 7H_2O$ was purchased from abcr GmbH (Karlsruhe, Germany). Ammonium hydroxide solution (28-30 wt% solution of NH_3 in water) was obtained from Acros Organics (Geel, Belgium). Other reagents were purchased from Sigma-Aldrich. All chemicals were of analytical grade and were used as received without any further purification. Water used for the preparation of aqueous solutions was purified using a Millipore-Q water purification system.

Synthesis

(1) Synthesis of NH_3-diffusion

An aqueous solution containing $CoSO_4 \cdot 7H_2O$ (0.124g) was prepared by dissolving the metal salts in deionized water (40 mL) in a beaker. After ultrasonic dissolution, the beaker was put into a sealed desiccator with 2 mL concentrated ammonium hydroxide solution. After the diffusion for 12 hours at room temperature, the precipitate was washed by centrifugation (9000 rpm, 5 min) with Milli-Q water two times, and dried at room temperature. The obtained nanomaterials were denoted as NH_3-diffusion in this paper.

(2) Synthesis of NaOH-slow

An aqueous solution containing $CoSO_4 \cdot 7H_2O$ (0.124g) was prepared by dissolving the metal salts in deionized water (40 mL) in a beaker. After ultrasonic dissolution, 2.5 mL NaOH solution (0.8 M) was added into $CoSO_4 \cdot 7H_2O$ solution via program-controlled dropwise addition (One drop every 120 to 180 seconds, with each drop being 40 to 60 μL). Then, the reaction solution was kept at room temperature for 12 hours. The precipitate was washed by centrifugation (9000 rpm, 5 min) with Milli-Q water two times, and dried at room temperature. The obtained nanomaterials were denoted as NaOH-slow in this paper.

(3) Synthesis of $NH_3 \cdot H_2O$-pour

An aqueous solution containing $CoSO_4 \cdot 7H_2O$ (0.124g) was prepared by dissolving the metal salts in deionized water (40 mL) in a beaker. Then, 2 mL of concentrated ammonia hydroxide solution was added to the above solution in one go. The reaction solution was kept at room temperature for 12 hours. The precipitate was washed by centrifugation (9000 rpm, 5 min) with Milli-Q water two times, and dried at room temperature. The obtained nanomaterials were denoted as $NH_3 \cdot H_2O$-pour in this paper.

(4) Synthesis of NaOH-pour-4.55:1-$CoSO_4 \cdot 7H_2O$

The synthetic procedure is similar to that of $NH_3 \cdot H_2O$-pour, except that 2.5 mL NaOH solution (0.8 M) was used as the base source. The obtained nanomaterials were denoted as NaOH-pour-4.55:1-$CoSO_4 \cdot 7H_2O$ in this paper.

(5) Synthesis of NaOH-pour-2.55:1-$CoSO_4 \cdot 7H_2O$/NaOH-pour-2:1-$CoSO_4 \cdot 7H_2O$/NaOH-pour-1:1-$CoSO_4 \cdot 7H_2O$

The synthetic procedure is similar to that of NaOH-pour-4.55:1-$CoSO_4 \cdot 7H_2O$, except that different volumes of NaOH solution (0.8 M) were used as the precursor solution.

Characterization

TEM images were acquired on a Zeiss Libra 120 microscope operating at 120 kV. TEM samples were prepared from ethanol dispersion or water solution on conventional TEM grids (carbon-coated copper grids, 400 mesh, supplied by Quantifoil GmbH). HAADF-STEM images were acquired on a Titan Themis microscope operated at 300 kV. EDX spectrum imaging was performed using a SuperX detector. The X-ray powder diffraction (XRD) measurements were carried out using a Rigaku SmartLab diffractometer having a Cu Kα rotating anode X-ray tube. The diffractometer is equipped with primary and secondary slit-optic, a 5-circle goniometer, and a HyPix3000 area detector. The measurements were done as a continuous symmetrical overview scan using a step size of $\Delta 2\Theta = 0.01°$, a count rate of 1deg/min, and a power setup of 45 kV/200 mA. The samples were mounted on a special low background Si sample holder due to the small amount of the available powder. The used SEM was a Zeiss CrossBeam 1540XB reaching a resolution of up to 1.1 nm at 20 kV and the acceleration voltage can be changed from 0.1 to 30 kV. It was equipped with an SE2 and an InLens detector. Attenuated-total-reflection-infrared spectroscopy (ATR-FTIR) was conducted on a Perkin Elmer device (Spectrum 100 FTIR, equipped with a diamond ATR crystal). HRTEM images were obtained on a JEOL-2010 microscope operated at 200 kV. The titration setup is a commercial, computer-controlled titration system manufactured by Metrohm. The titration software is Tiamo 2.2. UV-vis measurements were performed with an Ocean Optics USB-DT light source with an Ocean Optics USB2000+ spectrometer and a Varian Cary 50 BIO UV-vis spectrophotometer.

The Co K-edge XAFS characterizations were performed at the P65 beamline of PETRA III (Germany).*(1)* At the P65 beamline, the X-ray beam was generated using the 3rd-order harmonic radiation from an 11-period undulator. A water-cooled fixed exit Si(111) double crystal monochromator (DCM) was used to monochromatize the X-ray beam (energy resolution $\Delta E/E = 1.4 \times 10^{-4}$). The DCM was operated in QEXAFS mode, and the undulator offset to the DCM was calibrated to have the maximum photon flux. The X-ray beam was focused and collimated at the sample position to a spot size of approximately 1.0 mm × 0.5 mm (H × V). For the sample preparation, the sample powder was diluted with boron nitride to obtain an optimal concentration of Co and pressed into 13 mm diameter pellets. Co K-edge XAFS spectra from 7559 to 8709 eV with a step size of 0.5 eV were collected in transmission mode using ionization chamber detectors. XAFS spectra of a Co foil were also simultaneously collected using another ionization chamber detector during the measurements of the $Co(OH)_2$ samples. The energy of the incident beam was calibrated by assigning the energy of the first inflection in the first derivative XANES of Co foil to 7709 eV. Three repetition scans were collected for each sample and merged to improve the signal-to-noise ratio. The Co K-edge XAFS spectra were analyzed using the Demeter software package (including Athena and Artemis programs, version 0.9.26).*(2)* Pre-edge background subtraction and post-edge normalization of the XAFS data were performed using the Athena program. A linear regression background in the range of 7559 to 7679 eV) was determined, and a quadratic polynomial regression for post-edge normalization in the range of 7859 to 8709 eV was applied. The spectra were splined from k=0 Å^{-1} to k=16 Å^{-1} with rbkg of 1.2 and k-weight of 2. The fitting of EXAFS spectra (R-range: 1.2 to 3.2 Å, k-range: 3 to 11.5 Å^{-1}) was performed using the Artemis program based on scattering paths generated from FEFF6. The amplitude reduction factor S_0^2 was determined to be 0.75 by the fitting of k^2-weighted R-space EXAFS of Co foil based on the standard crystal parameters of Co foil and was used as a fixed parameter for the EXAFS fitting of other $Co(OH)_2$ samples.

Electrochemical Measurements

All the electrochemical tests were performed on the CHI-760E electrochemical workstation. Electrocatalytic oxygen evolution reaction performance was measured in the three-electrode system in 1 M KOH solution, in which Hg/HgO, carbon cloth, and carbon rod were used as the reference, working and counter electrodes, respectively. The electrocatalyst suspension was prepared by dispersing 10 mg electrocatalysts and 2 mg carbon black (VC-72) into the solution (1 mL) of water, isopropanol, and Nafion (v/v/v = 4/1/0.1). The mixed suspension was ultrasonicated for 1 hour to form a homogeneous electrocatalyst ink. Then 100 μL catalyst ink was pipetted on the carbon cloth with the coating area of $1 cm^2$ and dried with infrared light. All carbon cloth substrates needed to be pre-treated in acetone, ethanol, and water for 30 min before pasting. Linear sweep voltammetry curves were recorded in 1 M KOH solution at 5 mV s^{-1} with IR-100% compensation. For comparison, commercial RuO_2 electrocatalyst was measured under the same conditions.

All the potentials were converted to the reversible hydrogen electrode (RHE) by the Nernst equation:

$$E(RHE) = E(Hg/HgO) + 0.098 + 0.059 * pH$$

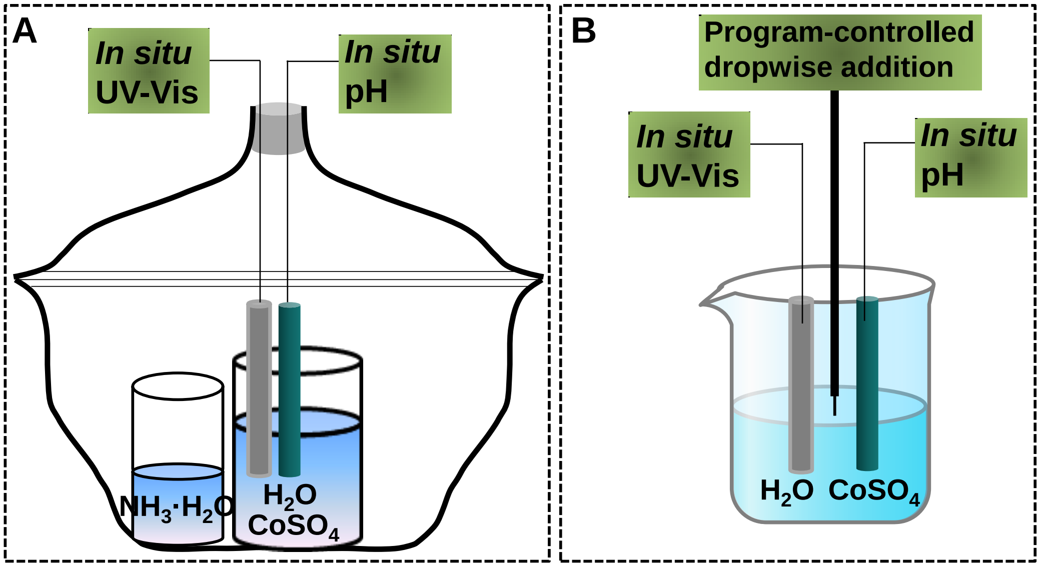

Figure S1. Schematic representation of set-ups used for the synthesis of (A) NH_3-diffusion and (B) NaOH-slow.

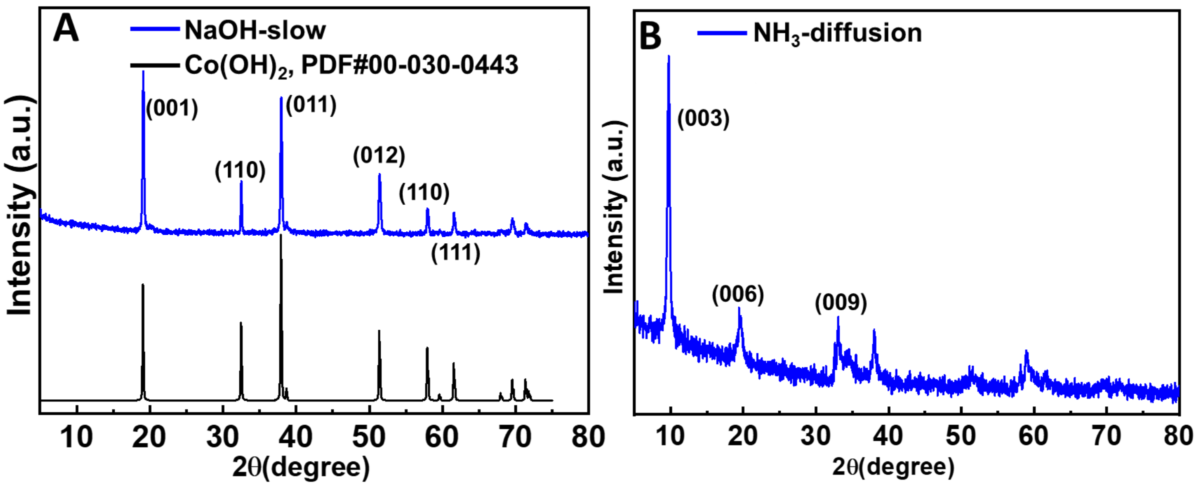

Figure S2. XRD patterns of (A) NaOH-slow and (B) NH_3-diffusion.

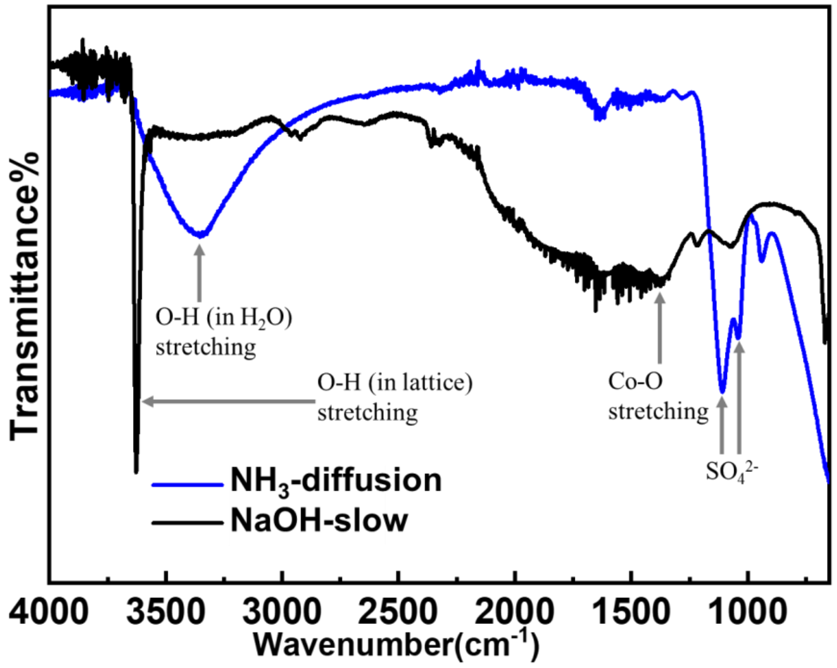

Figure S3. The FTIR spectrum of NaOH-slow and NH_3-diffusion. The sharp and strong absorption peaks at 1090 cm^{-1} and 1150 cm^{-1} are crucial evidence for the existence of SO_4^{2-} ions.*(3)*

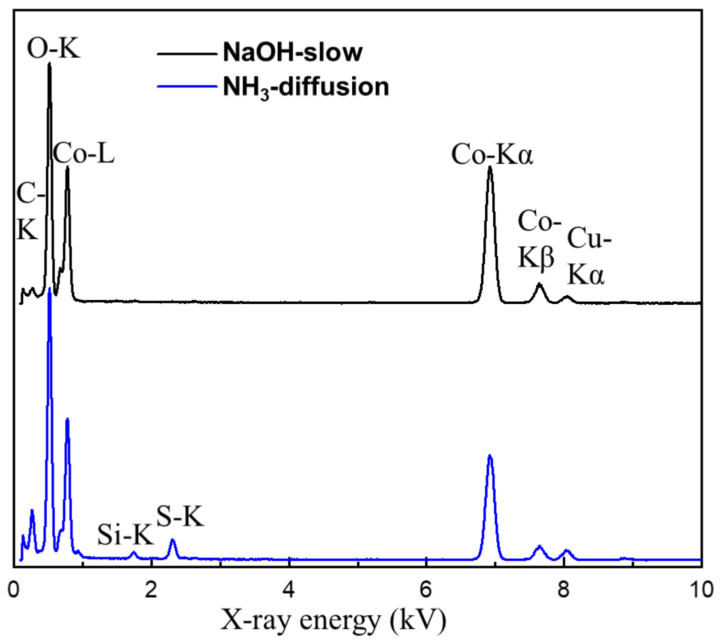

Figure S4. EDX spectrum of NaOH-slow and NH_3-diffusion.

Table S1 EXAFS fitting parameters at the Co K-edge for NH_3-diffusion and NaOH-slow.

Sample	Scattering path	C. N.	R [Å]	σ^2 [$Å^2$]	E_0 [eV]
NH_3-diffusion	Co-O	5.2 ±0.7	2.08 ± 0.01	0.0072 ± 0.0020	7719.4 ±1.1
	Co-Co	6.0 ±1.2	3.15 ± 0.01	0.0069 ± 0.0016	
NaOH-slow	Co-O	5.9 ±0.6	2.09 ± 0.01	0.0051 ± 0.0013	7718.3 ±0.8
	Co-Co	5.8 ±0.9	3.17 ± 0.01	0.0046 ± 0.0011	

C.N. = coordination number; R = interatomic distance; σ^2 = Debye-Waller factor; E_0 = E_0 position in the EXAFS fitting model.

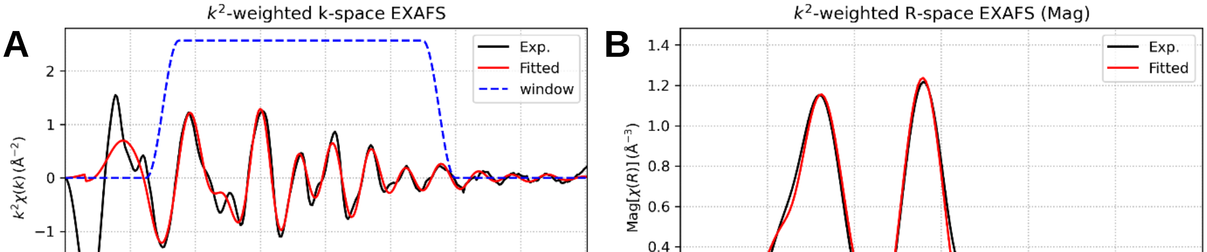

Figure S5. Fitting results of k^2-weighted k-space and R-space FT-EXAFS spectra of NH_3-diffusion (A-B) and NaOH-slow (C-D). The experimental EXAFS spectra and fitted spectra are plotted in black and red curves, respectively. The R-space spectra are plotted without phase correction.

Table S2. Possible scattering paths in the β-$Co(OH)_2$ structure.

**Scattering paths**	**C.N.**	**d [Å]**
Co-O(H)	6	2.097
Co-Co	6	3.173
Co-O(H)	6	3.803
Co-O(H)*	6	4.056
Co-Co*	2	4.640
Co-O(H)	12	4.953
Co-O(H)*	6	5

		.150
Co-Co	6	5<br>.496
Co-Co*	1<br>2	5<br>.621

β-$Co(OH)_2$ (space group: $P\bar{3}m1$) has a layered structure following the (001) facets. The scattering paths with * symbol in this table mean the center Co and the scatterer atoms are not within the same structural layer, and these scattering paths do not exist in the derived α-$Co(OH)_2$ structure as the gap between the layers are filled with other cations/anions. The structural parameters of the β-$Co(OH)_2$ are retieved from the Crystal Open Database (entry ID: 9009101).

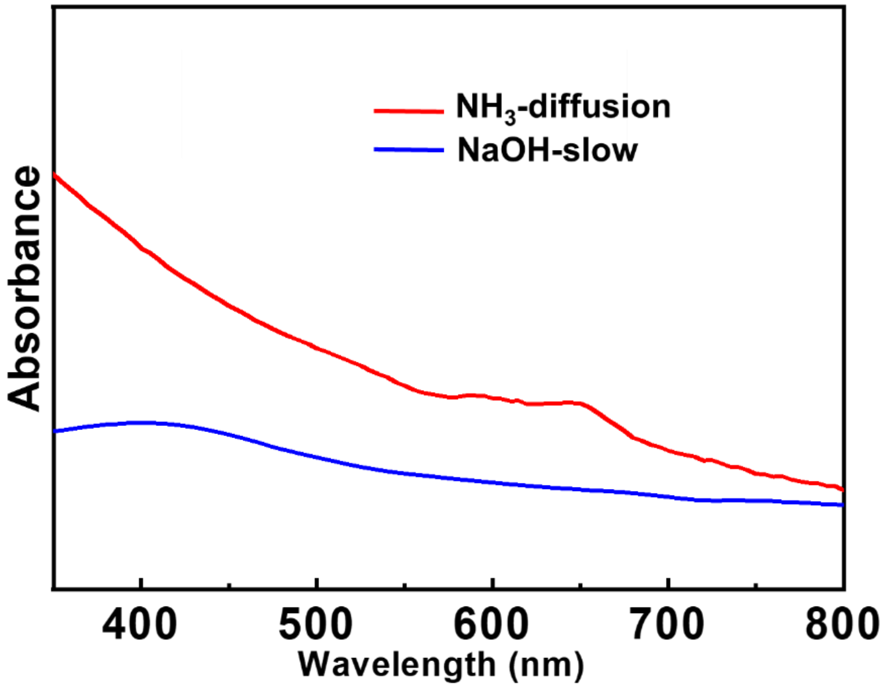

Figure S6. UV-Vis spectra of NH_3-diffusion and NaOH-slow.

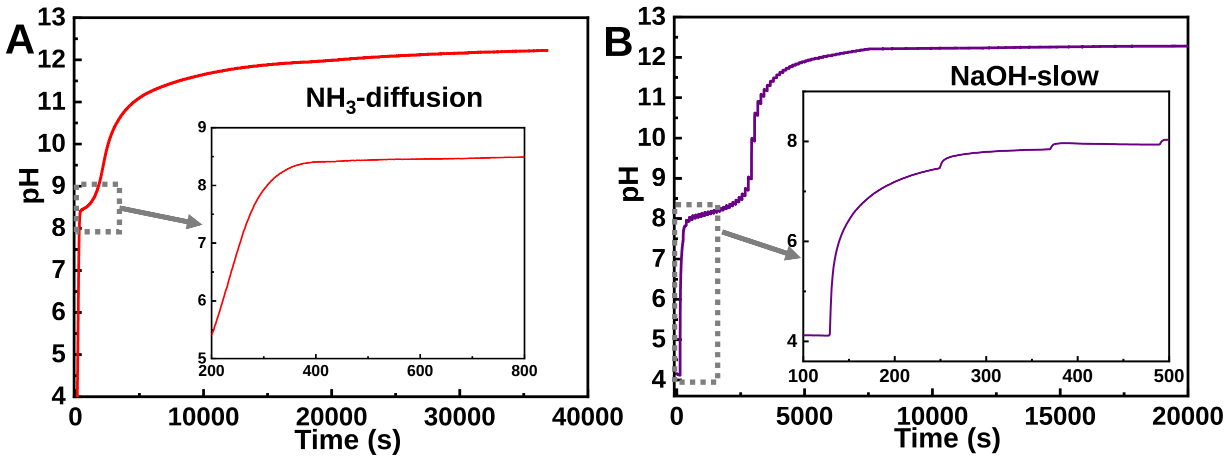

Figure S7. Observation of pH as a function of time for the reaction process of (A) NH_3-diffusion and (B) NaOH-slow.

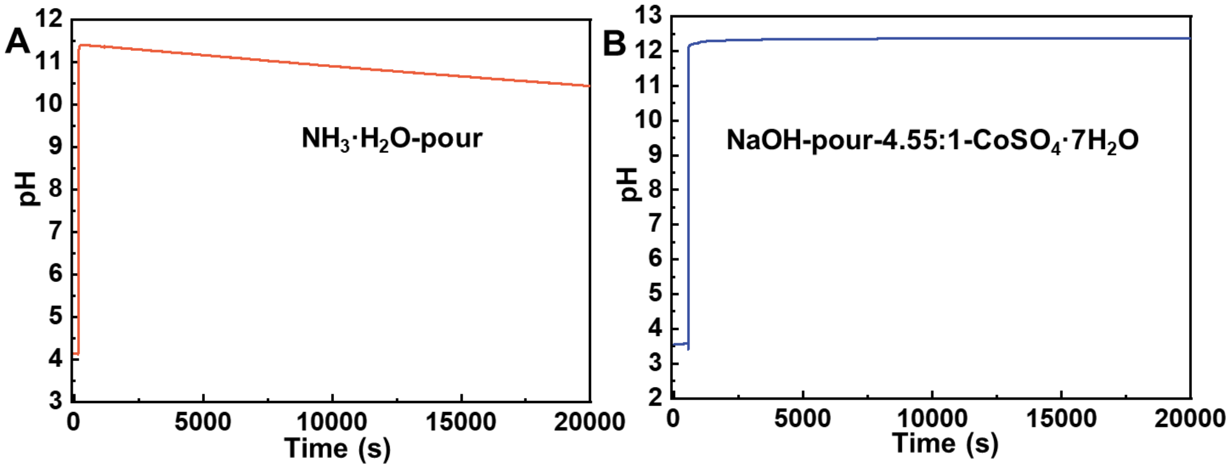

Figure S8. Observation of pH as a function of time for the reaction process of (A) $NH_3 \cdot H_2O$-pour and (B) NaOH-pour-4.55:1-$CoSO_4 \cdot 7H_2O$.

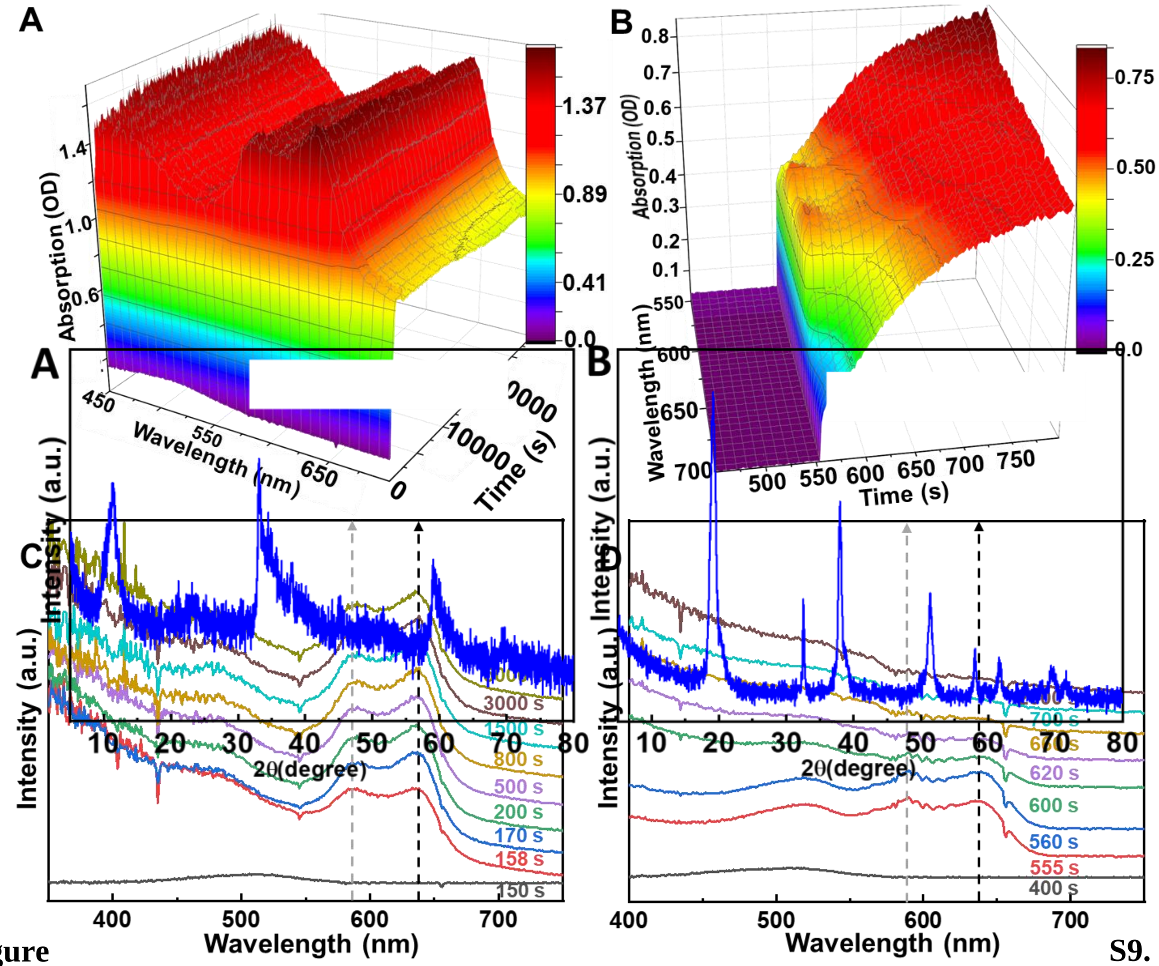

Figure S9. *In situ* UV-vis spectra as a function of immersion time for the reaction process of (A) $NH_3 \cdot H_2O$-pour and (B) NaOH-pour-4.55:1-$CoSO_4 \cdot 7H_2O$. UV-vis spectra at representative time points for the reaction process of (C) $NH_3 \cdot H_2O$-pour and (D) NaOH-pour-4.55:1-$CoSO_4 \cdot 7H_2O$.

Figure S10. XRD patterns of (A) $NH_3 \cdot H_2O$-pour and (B) NaOH-pour-4.55:1-$CoSO_4 \cdot 7H_2O$.

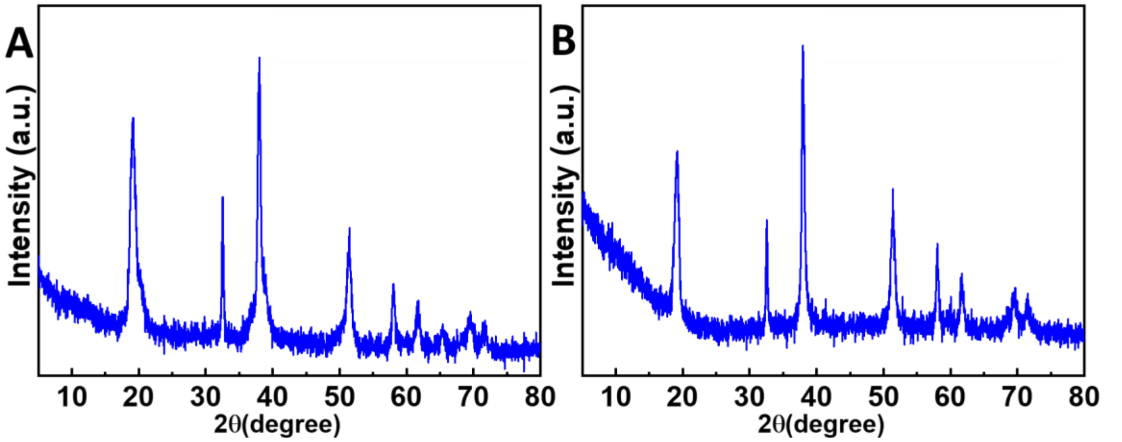

Figure S11. XRD patterns of (A) NaOH-pour-2.55:1-$CoSO_4 \cdot 7H_2O$ and (B) NaOH-pour-2:1-$CoSO_4 \cdot 7H_2O$.

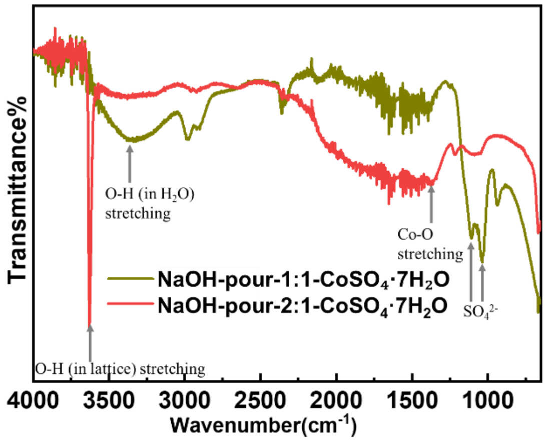

Figure S12. The FTIR spectrum of NaOH-pour-2:1-$CoSO_4 \cdot 7H_2O$ and NaOH-pour-1:1-$CoSO_4 \cdot 7H_2O$.

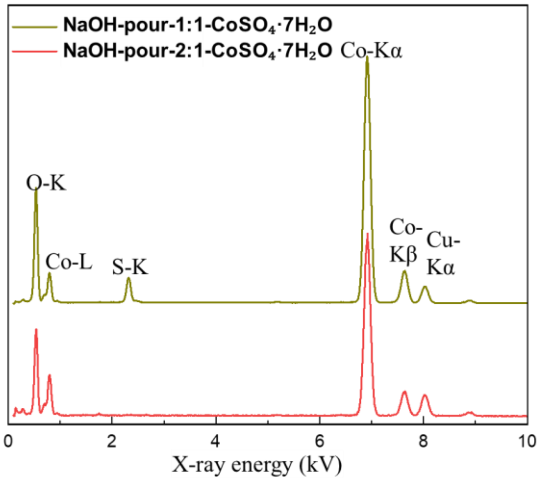

Figure S13. EDX spectrum of NaOH-pour-2:1-$CoSO_4 \cdot 7H_2O$ and NaOH-pour-1:1-$CoSO_4 \cdot 7H_2O$.

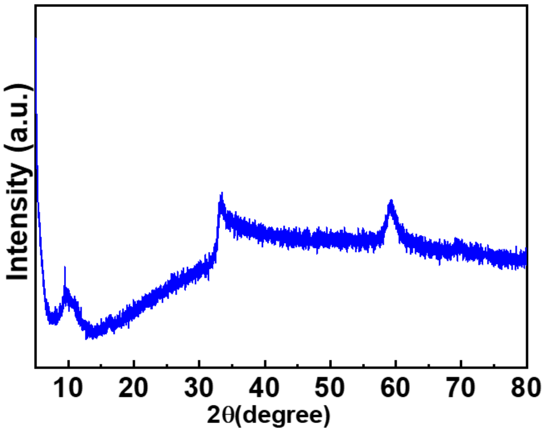

Figure S14. XRD pattern of NaOH-pour-1:1-$CoSO_4 \cdot 7H_2O$.

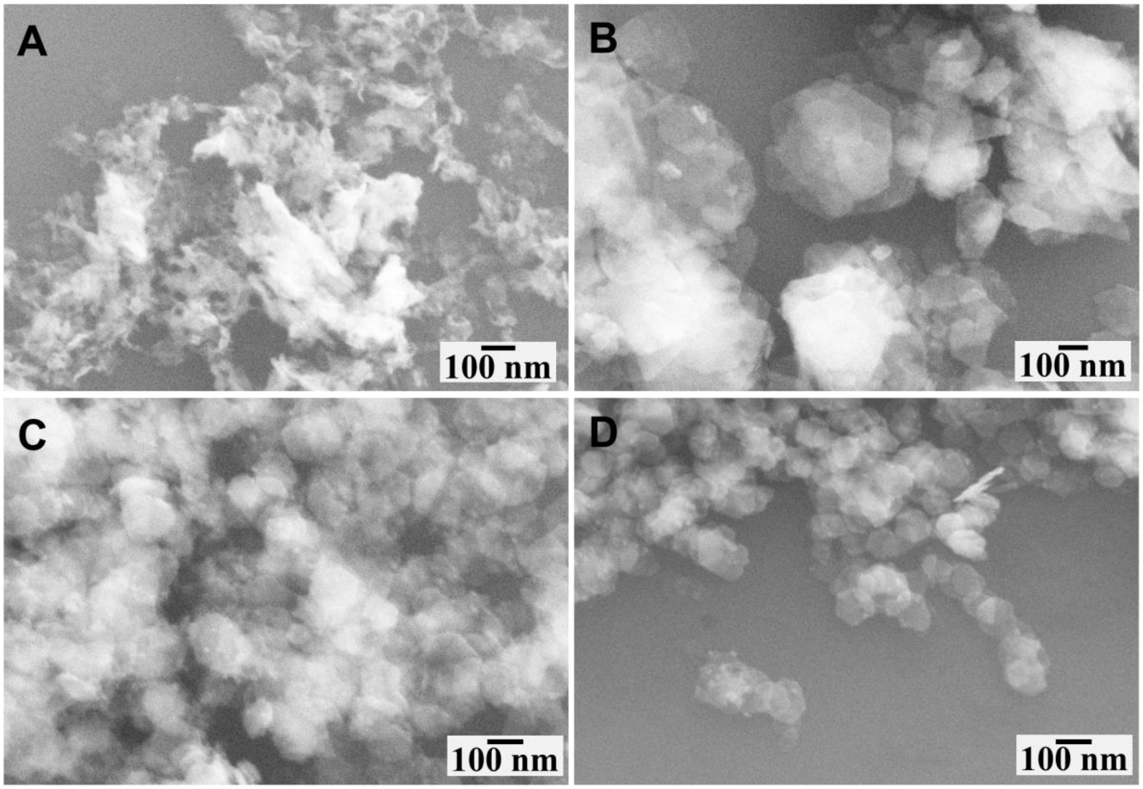

Figure S15. SEM images of (A) NaOH-pour-1:1-$CoSO_4 \cdot 7H_2O$, (B) NaOH-pour-2:1-$CoSO_4 \cdot 7H_2O$, (C) NaOH-pour-2.55:1-$CoSO_4 \cdot 7H_2O$ and (D) NaOH-pour-4.55:1-$CoSO_4 \cdot 7H_2O$.

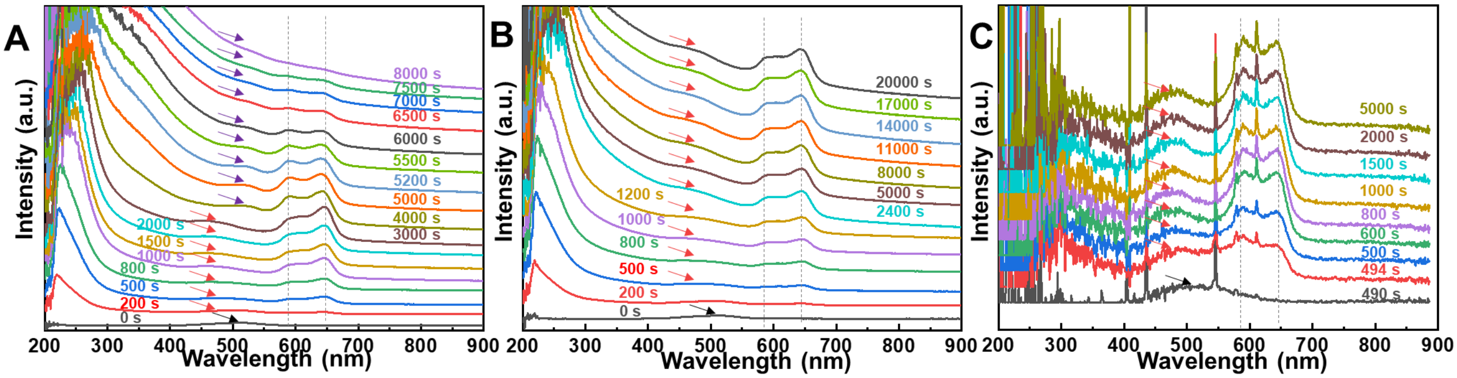

Figure S16. UV-vis spectra at representative time points for the reaction process of (A) NaOH-slow, (B) NH_3-diffusion and (C) NaOH-pour-1:1-$CoSO_4 \cdot 7H_2O$.

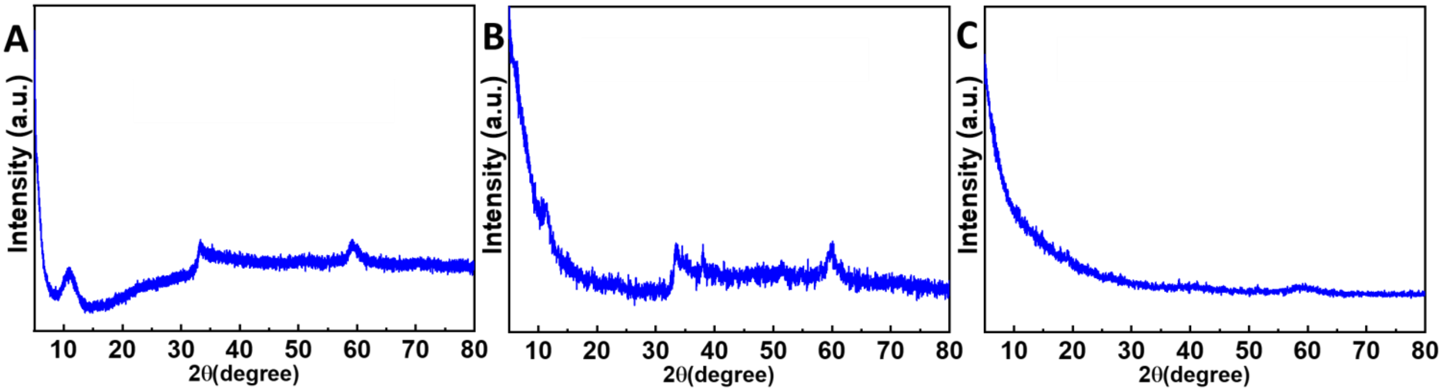

Figure S17. XRD patterns of (A) NaOH-pour-2.55:1-$CoSO_4 \cdot 7H_2O$-NH_4Cl, (B) NH_3-diffusion-NaOH-pour-2.55:1-$CoSO_4 \cdot 7H_2O$ and (C) NH_3-diffusion-clean-NaOH-pour-2.55:1-$CoSO_4 \cdot 7H_2O$.

Table S3 Comparison of catalytic parameters of NH_3-diffusion and other OER catalysts.

Catalyst	Electrolyte	Current density j (mA cm^{-2})	η（mV vs RHE）at corresponding j	Tafel slope (mV dec^{-1})	References
**NH_3-diffusion**	**1 M KOH**	**10**	**277**	**57.2**	**Our work**
GNRs/$Co(OH)_2$	1 M KOH	10	280	66	J. Mater. Sci., 2019, 54, 7692-7701
Amorphous nanostructured $Co(OH)_2$	1 M NaOH	10	360	56/122	J. Mater. Chem. A, 2016, 4, 991-999
Fe substituted α-$Co(OH)_2$	1 M KOH	10	295	52	J. Mater. Chem. A, 2017, 5, 1078-1084
Plasma-engraved Co_3O_4 nanosheets	0.1 M KOH	10	1530	68	Angew. Chem. Int. Ed. 2016, 55, 5277–5281
α-$Co(OH)_2$ nanomeshes	1 M KOH	10	303	69	Chem. Commun., 2018, 54, 4045-4048
Co_3O_4 nanosheet arrays on Ni foam	1 M KOH	100	303	75.6	Chem. Commun., 2018, 54, 12698-12701

CeO_2 Functionalized $Co(OH)_2$	1 M KOH	10	281	55	Small 2022, 18, 2107594
CoOOH topologically derived from $Co(OH)_2$	1 M KOH	10	426	60	Nat Commun, 2022, 13, 6650
$Co(OH)_2$ nanosheets	1 M KOH	10	292	87	Adv. Funct. Mater. 2022, 32, 2206407
Defect-rich α-$Co(OH)_2$	1 M KOH	10	307	56	ACS Appl. Eng. Mater. 2023, 1, 2698-2706
Cobalt silicate hydroxide	1 M KOH	10	301	88	Small 2024, 2401394
Ag-integrated $Co(OH)_2$	1 M KOH	10	253	53	New J. Chem., 2024, 48, 1671-1677

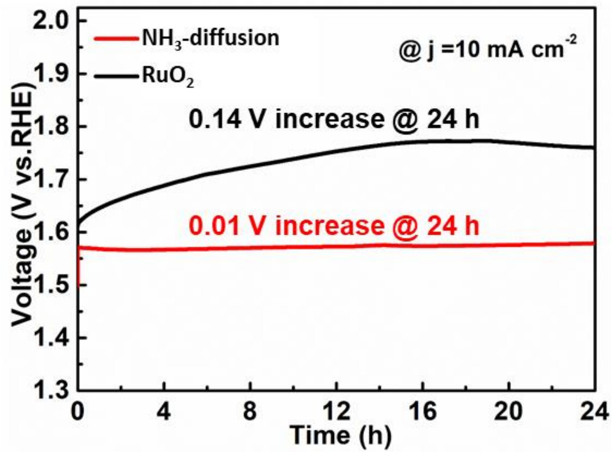

Figure S18. Long-term stability test of NH_3-diffusion and commercial RuO_2 electrocatalyst.

References

1. E. Welter, R. Chernikov, M. Herrmann, R. Nemausat, in AIP Conference Proceedings, *AIP Publishing*, **2054**, (2019).

2. B. Ravel, M. Newville, ATHENA, ARTEMIS, HEPHAESTUS: data analysis for X-ray absorption spectroscopy using IFEFFIT. *J. Synchrotron Rad*, **12**, 537-541 (2005).

3. F. X. Geng, H. Xin, Y. Matsushita, R. Z. Ma, M. Tanaka, F. Izumi, N. Iyi, T. Sasaki, New Layered rare-earth hydroxides with anion-exchange properties. *Chem. Eur. J.*, **14**, 9255-9260 (2008).
